# A Retrospective, Comparative Cohort Analysis of the Utilization of Voriconazole-Containing Regimens Versus Itraconazole in the Treatment of Blastomycosis

**DOI:** 10.3390/jof12040279

**Published:** 2026-04-14

**Authors:** Caitlin C. Schanz, Christina G. Rivera, Omar M. Abu Saleh, Josh Clement, Mark J. Enzler, Danielle Firkus, Kristin Cole, Paschalis Vergidis, Ryan W. Stevens

**Affiliations:** 1 Department of Pharmacy, Boston Medical Center, Boston, MA 02118, USA; 2 Department of Pharmacy, Mayo Clinic, Rochester, MN 55905, USA; 3 Division of Public Health, Infectious Diseases, and Occupational Medicine, Mayo Clinic, Rochester, MN 55905, USA; 4 Department of Pharmacy, New York Presbyterian Hospital, New York, NY 10065, USA; 5 Department of Pharmacy, Mayo Clinic Health System, Eau Claire, WI 54703, USA; 6 Department of Quantitative Health Sciences, Mayo Clinic, Rochester, MN 55905, USA

**Keywords:** itraconazole, voriconazole, blastomycosis, *Blastomyces*, antifungal agents, drug-related side effects, adverse reactions

## Abstract

Itraconazole is the recommended first-line antifungal agent in blastomycosis treatment. However, pharmacokinetic limitations and adverse drug events may necessitate the use of alternative antifungal agents. The primary objective of the study was to retrospectively describe a cohort of patients with blastomycosis who received voriconazole (as partial or complete therapy), for complete or partial treatment, compared to those who received itraconazole alone. Secondary objectives included rationale for voriconazole selection, treatment response, mortality, and adverse drug events. This retrospective multicenter cohort study included adult patients with proven/probable blastomycosis who received itraconazole or voriconazole. Treatment response was evaluated at one year or end of therapy, whichever came first. Mortality outcomes were assessed within 7 days of the last documented azole dose. Propensity score weighting and subgroup analysis were utilized to control confounding variables between cohorts. A total of 119 patients receiving itraconazole and 25 receiving voriconazole as complete or partial treatment were included. Voriconazole was often selected for CNS involvement or after intolerance to alternative azoles. After propensity score weighting, no significant difference in complete or partial treatment response was observed. Rates of all-cause mortality and blastomycosis-related mortality were numerically higher in the voriconazole cohort. Adverse drug event rates were similar between cohorts; however, discontinuation due to adverse events was more common in the voriconazole cohort. Voriconazole was most utilized for the treatment of blastomycosis in cases with CNS involvement or intolerance to other azoles. A statistically significant difference in response rate was not identified between voriconazole-containing or itraconazole-treated patients; however, given limited sample sizes, further data is needed to assess the equivalency of these agents in the treatment of blastomycosis.

## 1. Introduction

Blastomycosis is an infection caused by *Blastomyces*, a thermally dimorphic fungus found in soil and decaying organic material [1]. *Blastomyces dermatitidis* is responsible for most blastomycosis cases throughout North America and is endemic to the midwestern, southeastern, and south-central United States [2,3]. Additional species causing blastomycosis have been isolated in distinct geographic regions worldwide, including Africa and the Middle East [2]. Blastomycosis typically develops through inhalation of airborne conidia produced in the mold stage, leading to subsequent pulmonary infection in both immunocompromised and immunocompetent hosts [1]. Hematogenous dissemination of the yeast form occurs in up to 40% of cases including cutaneous, osteoarticular, genitourinary, and central nervous system (CNS) involvement [4].

Itraconazole is currently recommended as the first-line azole antifungal for treating mild-to-moderate blastomycosis or as definitive therapy following liposomal amphotericin B induction in severe or disseminated disease, according to guidelines from both the Infectious Diseases Society of America (IDSA) and the European Confederation of Medical Mycology (ECMM) [4,5]. The prominent position of this antifungal agent for this syndrome is supported by a phase 2 study showing itraconazole treatment success rates of 90–95% [6]. Yet, the ECMM guidelines also acknowledge the potential for use of alternative triazoles in the setting of azole intolerance, noting that evidence with other triazoles (i.e., voriconazole, posaconazole, isavuconazole, and fluconazole) is scarce.

The use of itraconazole in the treatment of blastomycosis, particularly when used for more than 6 months, can be complicated by various factors. Itraconazole has potential for serious toxicities, such as hepatotoxicity, QT prolongation, negative inotropic cardiac effects, and other cardiac toxicities [7]. The extent of oral bioavailability varies between formulations and depends on adherence to formulation-specific administration instructions [8]. A new formulation, suba-itraconazole, has demonstrated less absorption variability, but its use is limited due to cost and availability [9]. Failure to achieve therapeutic serum concentrations of itraconazole may lead clinicians to consider switching to an alternative azole for blastomycosis treatment [10].

An additional consideration in optimal azole selection for blastomycosis is the site of infection. Itraconazole demonstrates high protein binding and lipophilicity, resulting in cerebrospinal fluid (CSF) concentrations that are less than 10% of serum concentrations. While hydroxy-itraconazole has better distribution to the CSF, the concentration needed to predict a favorable response is unknown [11]. Thus, in the case of disseminated disease with CNS involvement, use of an alternative azole with superior CNS penetration, such as voriconazole, may be considered. Reported voriconazole CSF concentrations have ranged from 22% to 100% of serum concentrations. The increased CNS penetration compared to itraconazole may be attributed to voriconazole’s high bioavailability, moderate protein binding, and moderate lipophilicity [11]. Voriconazole has demonstrated in vitro activity against *B. dermatitidis* with minimum fungicidal concentrations (MFCs) of 0.125 mcg/mL and 4 mcg/mL in 50% and 90% of tested isolates, respectively [12]. Voriconazole is also associated with fewer formulation-related absorption concerns; however, active drug exposure may be dependent on patient *CYP2C19* metabolizer status [13].

This retrospective study aimed to describe the characteristics of patients with blastomycosis who received voriconazole, as complete or partial treatment, compared to those receiving itraconazole. Additionally, we attempted to summarize treatment response rates and adverse drug events between cohorts.

## 2. Patients and Methods

This multicenter, retrospective cohort study included adult patients (≥18 years of age) with proven/probable blastomycosis at any Mayo Clinic hospital or clinic in Minnesota, Wisconsin, Florida, or Arizona from 1 January 2000 to 31 December 2021. Proven blastomycosis was defined as clinical and/or radiographic evidence of infection and at least one of the following: isolation of *Blastomyces* in culture of respiratory, skin, or sterile specimen; histopathologic or cytopathologic evidence of yeast consistent with *Blastomyces*; or positive *Blastomyces* PCR. Probable blastomycosis was defined as clinical and/or radiographic evidence of infection and a positive serology (immunodiffusion or complement fixation) and/or urine or serum *Blastomyces* antigen. Patients were excluded if not treated with itraconazole or voriconazole, if they received less than 14 days or an unknown duration of azole antifungal therapy, if the primary syndrome was cutaneous blastomycosis without evidence of pulmonary or disseminated disease, if there was an unclear treatment response, if they were pregnant or incarcerated, or if they refused Minnesota research authorization per Minnesota Statute §144.295 (Minnesota residents only). Included patients were then categorized into itraconazole- or voriconazole-containing antifungal treatment regimens. Patients included in the voriconazole cohort received voriconazole for either complete or partial treatment of blastomycosis. The itraconazole cohort included patients not exposed to voriconazole at any point in the full duration of therapy. Disseminated disease was defined as evidence of extrapulmonary site involvement. CNS disease was defined as radiographic evidence of CNS involvement or positive Blastomyces antibodies, antigen, or culture growth from cerebrospinal fluid.

Baseline patient characteristics, antifungal treatment and duration, severity of disease, therapeutic drug monitoring, adverse drug events, and concentrations for both included agents were collected. Rationale for voriconazole use and transition between azole agents (when applicable) was collected from inpatient and outpatient provider documentation. Baseline patient and antifungal treatment characteristics were summarized using frequencies and percentages for categorical data and medians with interquartile ranges or means with standard deviations for continuous data. Comparisons between the itraconazole and voriconazole groups were made using Chi-square or Fisher’s exact tests for categorical data and either a Wilcoxon rank-sum test or *t*-test for continuous data.

Secondary outcomes included treatment response, all-cause mortality, mortality due to blastomycosis, occurrence of specific adverse drug events, adverse drug event rates, and discontinuation of azole treatment related to adverse drug events. Treatment responses were defined as follows: complete (resolution of all clinical symptoms and radiographic abnormalities), partial (≥50% improvement in radiographic abnormalities and clinical improvement), stable (<50% improvement in radiographic abnormalities and no clinical improvement), and failure (worsening of clinical manifestations or death due to blastomycosis) [10]. Partial or complete treatment response was evaluated at one year or the end of antifungal therapy, whichever came first. Outcomes were adjudicated by two Infectious Diseases physicians, with a third Infectious Diseases physician adjudication if disagreement arose between the first two reviewers. Mortality outcomes were evaluated within 7 days of the last documented administration of azole therapy. Mortality secondary to blastomycosis was determined by documented autopsy reports or by the aforementioned physician adjudication process if autopsy results were unavailable or indeterminate. Adverse drug events were attributed to the specific azole being administered at the time of the event regardless of treatment arm.

For the outcome analysis, power was determined based on an estimated 86% expected complete response rate [14]. Therefore, 80 itraconazole and 30 voriconazole patients would have 80% power to detect a difference of 19% or more between groups. Due to the hypothesized imbalance in patient and clinical characteristics between treatment cohorts, a propensity score weighted version of the analyses was also performed. Propensity scoring variables were determined a priori and included age, gender, body mass index, Charlson comorbidity index, severity of disease, requirement for mechanical ventilation, disseminated blastomycoses, CNS involvement, and amphotericin B use. Statistical analysis was completed with SAS version 9.4 software (SAS Institute, Inc.; Cary, NC, USA). This study was approved by the Mayo Clinic IRB (IRB #19-011153).

## 3. Results

**Figure 1:**
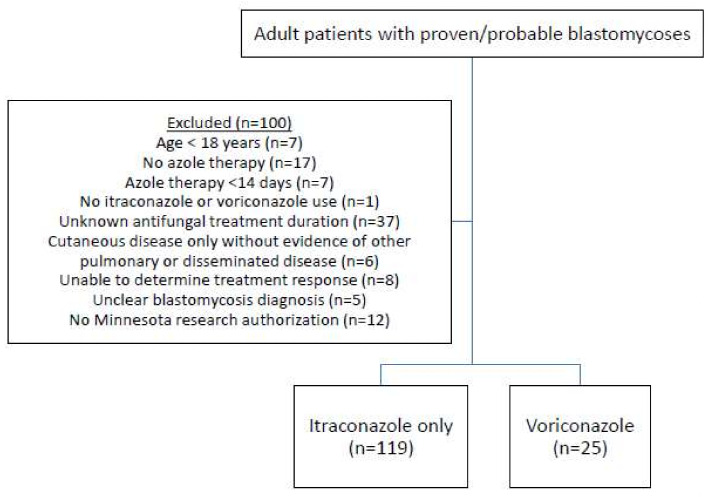
Patient Inclusion Flow Diagram.

A total of 244 patients diagnosed with blastomycosis from 1 January 2000 to 31 December 2021 were evaluated for inclusion. One hundred of these patients were excluded based on pre-defined criteria (Figure 1). The included cohort (*n* = 144) comprised 119 (83%) patients who received itraconazole and 25 (17%) patients who received voriconazole as complete or partial azole treatment. Of the included patients, 128 (88.9%) had proven disease and 16 (11.1%) had probable disease. The most common laboratory findings in those with proven disease were culture growth (*n* = 100) followed by identification on pathology (*n* = 26). Only 2 patients with proven disease were classified as such based on PCR alone. For those with probable disease, laboratory findings included antibodies or antigen alone in 9 and 7 patients, respectively.

### 3.1. Cohort Characteristics

Baseline patient characteristics between itraconazole and voriconazole cohorts are presented in Table 1. In the unweighted analysis, the mean age was comparable in both cohorts. Patients were predominantly of white race and male sex. Differences were observed between the cohorts in the level of care provided, with the voriconazole group having higher rates of hospitalization, including intensive care unit admissions (*p* = 0.009). Both treatment arms had similar durations of azole antifungals and total antifungal treatment (Table 2). A total of 55 (38%) patients received amphotericin B in addition to an azole antifungal: 37 (31%) in the itraconazole cohort and 18 (72%) in the voriconazole cohort (*p* < 0.001). Median duration of amphotericin B treatment was also significantly longer in the voriconazole group (20 days vs. 12 days, *p* = 0.026). Itraconazole parent and combined itraconazole/hydroxy-itraconazole serum concentrations were collected in 75 patients in the itraconazole group resulting median concentrations of 1 mcg/mL (IQR 0.7–1.6 mcg/mL) and 2.6 mcg/mL (IQR 1.7–4 mcg/mL), respectively. Voriconazole serum concentrations were collected in 18 of 25 patients in the voriconazole cohort, resulting in a median concentration of 2.3 mcg/mL (IQR 1.2–3.1). The voriconazole cohort was found to have higher rates of disseminated disease (52% vs. 25%, *p* = 0.008) and CNS involvement (28% vs. 1%, *p* < 0.001) compared to the itraconazole cohort.

Within the voriconazole cohort, 4 (16%) patients received only voriconazole for the total duration of azole therapy, 10 (40%) received voriconazole for ≥50% of azole therapy, and 11 (44%) received voriconazole for <50% of the total duration of azole therapy (Table 2). The median duration of therapy in each of these voriconazole groups was 348, 225, and 21 days, respectively. In the cohort who received voriconazole as partial therapy, 18 (72%) patients also received itraconazole either prior to or after voriconazole administration. Of note, 8 patients from the full cohort exhibited CNS involvement. Seven of these received voriconazole, 6 of which received the drug for ≥50% of the treatment duration.

Within the overall voriconazole cohort, 12 (48%) patients received voriconazole as the first azole in treatment. Voriconazole was mostly commonly selected for use as initial therapy due to CNS involvement/penetration or desire to empirically cover for alternative fungal pathogens (Table 3). Eight of these patients later transitioned to an alternate antifungal with the most common justification for transition away from voriconazole being the provider’s desire to shift to itraconazole given its standing as preferred therapy in blastomycosis.

Thirteen patients (52%) from the overall voriconazole cohort received the agent after administration of an alternative azole antifungal (Table 3). Most patients transitioned to voriconazole as an alternative agent due to experiencing adverse drug events to other azoles. Of these 13 patients, eight would go on to be transitioned from voriconazole to an alternative azole later in therapy, primarily due to occurrence of voriconazole related adverse drug events (*n* = 7/8, 87.5%).

### 3.2. Outcomes Analysis

In the unweighted cohort comparison, high rates of complete or partial treatment response were seen with both itraconazole and voriconazole (94% vs. 92%, *p* = 0.66). Numerically higher rates of all-cause mortality (6% vs. 16%, *p* = 0.099) and mortality due to blastomycosis (3% vs. 8%, *p* = 0.21) within 7 days of the last documented dose of azole were observed in the voriconazole cohort but these differences were not statistically significant (Table 4). Rates of adverse drug events were high but similar with use of itraconazole and voriconazole (45% vs. 48%, *p* = 0.80); however, a higher rate of discontinuation of azole therapy due to adverse drug events was observed with voriconazole use (12% vs. 32%, *p* = 0.015). Dermatological effects and visual disturbances were observed at significantly higher rates with voriconazole (Table 5).

After propensity score weighting, there was no significant difference in complete or partial treatment response, all-cause mortality, or mortality due to blastomycosis between the cohorts (Table 4). These findings also remained consistent even when excluding patients who received <50% voriconazole (Supplemental Table S1). Rates of adverse drug events mirrored the findings of the unweighted cohort, with a higher rate of azole discontinuation due to adverse drug events observed with voriconazole (Table 5).

## 4. Discussion

Our retrospective study demonstrated that voriconazole was primarily utilized when alternative azole therapy was not tolerated or if there was probable or proven blastomycosis involvement of the CNS. Though the voriconazole-containing regimen cohort had higher rates of disseminated/CNS diseases, there was no significant difference in terms of complete/partial response to therapy when compared to the itraconazole cohort. After accounting for baseline differences between groups with propensity score weighting, the findings remained consistent, showing similar rates of partial or complete treatment response. Rates of all-cause mortality and mortality due to blastomycosis were numerically, but not statistically, higher in the voriconazole-containing regimen cohort, including after propensity score matching. Additionally, while the overall rates of adverse drug events were similar between cohorts, the voriconazole-containing regimen cohort exhibited a higher rate of therapy discontinuation secondary to adverse drug events compared to the itraconazole group.

Previous literature has evaluated the use of itraconazole in the treatment of endemic mycoses. In a small, prospective cohort study, 43 of 48 patients with blastomycosis demonstrated a successful treatment response with itraconazole [6]. In a recent retrospective cohort study, voriconazole was compared to itraconazole for initial step-down treatment of histoplasmosis. Results suggested that patients receiving voriconazole had higher mortality rates during the first 42 days after the initiation of antifungal treatment compared to itraconazole; however, this difference was not observed between 42 and 180 days [14]. The higher mortality rate associated with voriconazole in histoplasmosis may be partially attributable to mutations in lanosterol 14-α-demethylase, leading to possible resistance to short chain-azoles, such as voriconazole and fluconazole [15]. However, such mutations have not been reported with blastomycosis. This previous analysis of azole therapy in histoplasmosis only included patients who received voriconazole as the initial azole antifungal therapy with or without amphotericin B induction. Our study differs in that patients who received voriconazole at any point during their treatment period were included, rather than just those receiving the agent as initial therapy; however, we acknowledge that initial azole selection may play a role in overall outcomes. Yet, our approach offers a more comprehensive inclusion of voriconazole as utilized in real-world clinical scenarios, especially as an alternative when itraconazole is not tolerated.

Data pertaining to use of alternative azole antifungals in blastomycosis is limited. In a case report of eight patients who exhibited disseminated blastomycosis with CNS involvement, seven patients that received amphotericin B followed by voriconazole had favorable outcomes [16]. While case reports and series have suggested success with voriconazole, this, to our knowledge, is the largest study that describes voriconazole utilization trends and attempts to analyze outcomes in the treatment of blastomycosis, offering a broader perspective on relative safety and efficacy [16,17,18,19]. Additionally, Scolarici et al. reported the use of isavuconazole in the treatment of 14 patients with blastomycosis, seven of which had CNS involvement, with a clinical cure rate of 79% [20].

Overall drug event rates were high in both the voriconazole-containing and itraconazole groups in this study. Other works have demonstrated similar rates of cumulative adverse drug event occurrence with azole antifungals. A previous pharmacovigilance study reported a total rate of azole adverse drug events of up to ~40% [21]. In our study, voriconazole exhibited higher rates of dermatologic effects and ocular or visual disturbances. These adverse drug events have commonly been previously reported with voriconazole and may be related to its higher rate of distribution to the CNS [22]. Additionally, the higher rate of ocular or visual disturbances with voriconazole may be related to its CNS distribution. The high rate of adverse drug events in both cohorts, coupled with the higher rate of therapy discontinuation due to adverse drug events in the voriconazole group, underscore the importance of careful monitoring and management of adverse drug events when utilizing either itraconazole or voriconazole for blastomycosis.

Limitations of the study include small sample size, imbalance of total patients in each treatment arm, and key differences in cohorts (e.g., differences in disseminated disease and CNS involvement). Due to itraconazole being recommended as the first-line azole antifungal per guideline recommendations, it is not unexpected that few patients received voriconazole as partial or complete treatment for blastomycosis. Inclusion of patients receiving partial voriconazole regimens in the voriconazole cohort allowed for some in the voriconazole cohort to also have received itraconazole. When assessing clinical outcomes and acknowledging the imbalance between the unweighted voriconazole-containing and itraconazole cohorts, propensity score matching was utilized. Some of the observed cohort differences, even after propensity matching, may limit the interpretation of clinical outcome findings. Particularly, disease severity in the voriconazole-containing cohort may have played a role in the observed mortality rates, as patients who received voriconazole had higher rates of disseminated disease, CNS involvement, hospitalization, and more often required amphotericin B administration compared to the itraconazole cohort. In an attempt to further minimize cohort heterogeneity, a subgroup analysis was performed including only those that received at least 50% of the total regimen as voriconazole; however, with the limited overall enrollment, sufficient power was unlikely reached to confidently assess differences in clinical findings or adverse drug event rates. Finally, this study is limited by its retrospective design, wherein treatment outcomes and adverse drug events were reliant on access to imaging and clear documentation. While high rates of adverse drug events were documented with both itraconazole and voriconazole in this study, the overall rates may be underestimated due to lack of adverse effect documentation in electronic health records and/or potential for related presentations to outside healthcare organizations.

Limitations considered, this study provides additional data to clinicians who may be considering the use of voriconazole for treatment of blastomycosis when itraconazole utilization is not feasible or contraindicated. Given limited enrollment in our study, further research with a larger sample size is needed to explore potential differences in mortality, treatment response, and adverse drug events between these azoles when utilized in management of blastomycosis.

While itraconazole is recognized as the first-line oral azole antifungal for treatment of blastomycosis, the results of this study suggest that voriconazole has been used in clinical practice when itraconazole is not tolerated or there is concern for CNS involvement. Treatment response and adverse drug events were similar between cohorts, but mortality was numerically, though not statistically, more common in the voriconazole cohort. Voriconazole treatment was also associated with a higher rate of azole discontinuation due to adverse drug events.

## Figures and Tables

**Table 1:** Baseline Patient Characteristics and Disease Severity.

	Unweighted Cohort			Weighted Cohort **		
	Itraconazole (*n* = 119)	Voriconazole (*n* = 25)	*p*-Value	Itraconazole (*n* = 119)	Voriconazole (*n* = 25)	*p*-Value
Age at blastomycosis diagnosis, Mean (SD)	50.5 (17.3)	52.4 (17.5)	0.53	51.1 (17.4)	52.1 (25.7)	0.79
Sex			0.91			0.91
Male	87 (73%)	18 (72%)		72%	73%	
Female	32 (27%)	7 (28%)		28%	27%	
Race			0.74			0.63
White	102 (86%)	24 (96%)		86%	99%	
American Indian/Alaskan native	2 (2%)	1 (4%)		2%	1%	
Asian	6 (5%)	0 (0%)		5%	0%	
Black or African American	3 (3%)	0 (0%)		2%	0%	
Other	4 (3%)	0 (0%)		3%	0%	
Unknown	2 (2%)	0 (0%)		2%	0%	
Ethnicity			0.44			0.29
Hispanic or Latino or Mexican	3 (3%)	1 (4%)		3%	1%	
Non-Hispanic or Latino	104 (87%)	23 (92%)		87%	98%	
Unknown	12 (10%)	1 (4%)		11%	1%	
Height (in cm), Mean (SD)	173.9 (9.0)	175.3 (12.1)	0.60	173.8 (8.9)	175.1 (12.4)	0.52
Weight (in kg), Median (IQR)	87 (74, 99)	87 (70, 97)	0.98	87 (74, 99)	88 (71, 99)	0.50
BMI (in kg/m^2^), Median (IQR)	28 (24, 32)	29 (23, 34)	0.83	28 (24, 32)	30 (23, 34)	0.60
Charlson Index, Median (IQR)	1 (0, 3)	3 (0, 4)	0.098	1 (0, 3)	1 (0, 3)	0.70
Severity of Disease			0.009			0.46
No hospitalization	61 (51%)	6 (24%)		47%	33%	
Hospitalized	47 (39%)	12 (48%)		41%	51%	
Intensive care unit	11 (9%)	7 (28%)		13%	16%	
Mechanical ventilation	9 (8%)	3 (12%)	0.44	9%	10%	0.84
ECMO	3 (3%)	0 (0%)	0.99	3%	0%	0.99
Disseminated blastomycoses	30 (25%)	13 (52%)	0.008	28%	42%	0.15
CNS involvement	1 (1%)	7 (28%)	<0.001	2%	8%	0.15

ECMO: Extracorporeal membrane oxygenation, CNS: Central nervous system. ** Results are from the hypothetical cohort created using inverse probability weighting to try and eliminate selection bias.

**Table 2:** Antifungal Treatment.

	Unweighted Cohort			Weighted Cohort *		
	Itraconazole (*n* = 119)	Voriconazole (*n* = 25)	*p*-Value	Itraconazole (*n* = 119)	Voriconazole (*n* = 25)	*p*-Value
Total duration of antifungal treatment in days, Median (IQR)	273 (187, 365)	356 (233, 365)	0.27	273 (189, 357)	308 (106, 359)	0.74
Total duration of azole treatment in days, Median (IQR)	273 (186, 353)	343 (205, 356)	0.60	272 (186, 352)	311 (104, 360)	0.66
Amphotericin B induction	37 (31%)	18 (72%)	<0.001	35%	45%	0.35
Amphotericin B duration (days), Median (IQR)	12 (7, 15)	20 (13, 32)	0.026	12 (7, 17)	18 (11, 31)	0.053
Amphotericin B duration unknown	3 (8%)	0 (0%)	0.54	6%	0%	0.86
Azole antifungals used						
Itraconazole	119 (100%)	18 (72%)	<0.001	100%	89%	<0.001
Voriconazole	0 (0%)	25 (100%)	<0.001	0%	100%	<0.001
Posaconazole	3 (3%)	3 (12%)	0.031	3%	5%	0.56
Fluconazole	7 (6%)	3 (12%)	0.27	6%	12%	0.27
Isavuconazole	1 (1%)	0 (0%)	0.65	1%	0%	0.65
First azole antifungal used			<0.001			<0.001
Itraconazole	115 (97%)	11 (44%)		97%	58%	
Voriconazole	0 (0%)	12 (48%)		0%	35%	
Posaconazole	0 (0%)	0 (0%)		0%	0%	
Fluconazole	3 (3%)	2 (8%)		3%	7%	
Isavuconazole	1 (1%)	0 (0%)		1%	0%	
% voriconazole of azole treatment						
Only voriconazole	---	4 (16%)	---	---	4%	---
<50% voriconazole	---	11 (44%)	---	---	58%	---
≥50% voriconazole	---	10 (40%)	---	---	38%	---
Duration of voriconazole (days), Median (IQR)						
Only voriconazole	---	348 (340, 357)	---	---	346 (333, 349)	---
<50% voriconazole	---	21 (8, 61)	---	---	19 (7, 34)	---
≥50% voriconazole	---	225 (142, 266)	---	---	186 (52, 267)	---

* Results are from the hypothetical cohort created using inverse probability weighting to try and eliminate selection bias.

**Table 3:** Rationale for Voriconazole use in Patients that Received any-duration Voriconazole.

	Total Cohort (*n* = 25)	Voriconazole as First Azole (*n* = 12)	Voriconazole Utilized NOT as First Azole (*n* = 13)
**Rationale for voriconazole use**			
CNS involvement/penetration	7 (28%)	6 (50%)	1 (7.69%)
Empiric coverage of alternative pathogens	4 (16%)	3 (25%)	1 (7.69%)
Availability of IV formulation	2 (8%)	1 (8.33%)	1 (7.69%)
Unknown	3 (12%)	2 (16.67%)	1 (7.69%)
Adverse drug event to other azoles	8 (32%)	---	8 (61.54%)
Inability to achieve therapeutic serum concentrations with itraconazole	1 (4%)	---	1 (7.69%)
Voriconazole transitioned to alternative antifungal during therapy course	16 (64%)	8 (66.67%)	8 (61.54%)
**Rationale for discontinuation of voriconazole therapy if transitioned off therapy**			
Adverse drug reaction to voriconazole	8 (32%)	1 (12.5%)	7 (87.5%)
Transition to preferred therapy (itraconazole)	7 (28%)	6 (75%)	1 (12.5%)
Inability to achieve therapeutic serum concentrations with voriconazole	1 (4%)	1 (12.5%)	0 (0%)

**Table 4:** Treatment Response.

	Unweighted Cohort			Weighted Cohort *		
	Itraconazole (*n* = 119)	Voriconazole (*n* = 25)	*p*-Value	Itraconazole (*n* = 119)	Voriconazole (*n* = 25)	*p*-Value
Complete/Partial treatment response	112 (94%)	23 (92%)	0.66	94%	91%	0.67
All-cause mortality	7 (6%)	4 (16%)	0.099	8%	17%	0.14
Mortality due to blastomycosis	3 (3%)	2 (8%)	0.21	3%	9%	0.21

* Results are from the hypothetical cohort created using inverse probability weighting to try and eliminate selection bias.

**Table 5:** Safety Outcomes.

	Unweighted Cohort			Weighted Cohort *		
	Itraconazole (*n* = 137)	Voriconazole (*n* = 25)	*p*-Value	Itraconazole (*n* = 141)	Voriconazole (*n* = 25)	*p*-Value
Any Adverse Event	62 (45%)	12 (48%)	0.80	45%	50%	0.66
QTc prolongation	2 (1%)	0 (0%)	0.99	2%	0%	0.43
Liver Dysfunction	11 (8%)	4 (16%)	0.25	8%	24%	0.011
Inotropic Toxicity	9 (7%)	0 (0%)	0.36	7%	0%	0.16
Edema only	13 (10%)	0 (0%)	0.22	9%	0%	0.11
Dermatologic effects	6 (4%)	5 (20%)	0.004	4%	21%	<0.001
Hypertriglyceridemia	0 (0%)	0 (0%)	---	0%	0%	---
GI intolerance	22 (16%)	1 (4%)	0.21	16%	9%	0.33
Headache	6 (4%)	0 (0%)	0.59	4%	0%	0.30
Respiratory effects	2 (1%)	0 (0%)	0.99	2%	0%	0.46
New onset hypertension	4 (3%)	0 (0%)	0.99	3%	0%	0.39
New onset hypokalemia	3 (2%)	0 (0%)	0.99	2%	0%	0.45
Alopecia	0 (0%)	0 (0%)	---	0%	0%	---
Tremor	2 (1%)	0 (0%)	0.99	1%	0%	0.72
Ocular/visual disturbances	2 (1%)	4 (16%)	0.006	1%	12%	0.004
Other	5 (4%)	2 (8%)	0.29	3%	4%	0.89
Adverse effect leading to discontinuation of azole	16 (12%)	8 (32%)	0.015	13%	34%	0.013

* Results are from the hypothetical cohort created using inverse probability weighting to try and eliminate selection bias.

## Data Availability

The raw data supporting the conclusions of this article will be made available by the authors on request.

## Supplementary Materials

The following supporting information can be downloaded at: https://www.mdpi.com/article/10.3390/jof12040279/s1, Table S1: Treatment Response Excluding <50% of Voriconazole Treatment.
