# Supplementary material for: A Retrospective, Comparative Cohort Analysis of the Utilization of Voriconazole-Containing Regimens Versus Itraconazole in the Treatment of Blastomycosis
Source: J Fungi (Basel). 2026 Apr 14;12(4):279. doi: 10.3390/jof12040279 (PMC13117282; doi:10.3390/jof12040279)
Supplement: Supplementary file 1 [file jof-12-00279-s001.zip › jof-4233196-supplementary.pdf]

Supplemental Table S1: Treatment Response Excluding <50% of Voriconazole Treatment

	Unweighted Cohort			Weighted Cohort		
	Itraconazole (N = 119)	Voriconazole (N = 14)	P-value	Itraconazole (N = 119)	Voriconazole (N = 14)	P-value
Treatment response			>0.99			0.76
Complete/Partial	112 (94%)	13 (93%)		94%	96%	
Failure/Stable	7 (6%)	1 (7%)		6%	4%	
All-cause mortality	7 (6%)	2 (14%)	0.24	7%	19%	0.12
Mortality due to blastomycosis	3 (3%)	1 (7%)	0.36	3%	4%	0.80
